# Nest-site selection and its influence on breeding success in a poorly-known and declining seabird: The Tahiti petrel *Pseudobulweria rostrata*

**DOI:** 10.1371/journal.pone.0267408

**Published:** 2022-04-27

**Authors:** Angélique Pagenaud, Andreas Ravache, Karen Bourgeois, Mathieu Mathivet, Édouard Bourguet, Éric Vidal, Martin Thibault

**Affiliations:** 1 UMR ENTROPIE, IRD, IFREMER, CNRS, University of Reunion, University of New Caledonia, Noumea, New Caledonia; 2 University of Aix-Marseille, CNRS, IRD, University of Avignon, IMBE, Centre IRD, Noumea, New Caledonia; 3 EB Expertise, Noumea, New Caledonia; MARE – Marine and Environmental Sciences Centre, PORTUGAL

## Abstract

The Tahiti petrel (*Pseudobulweria rostrata*) is a rare and declining seabird whose breeding biology and nest-site selection are poorly known. Nest-site selection is critical to seabird population fitness, and understanding the factors driving it is essential for designing effective conservation measures. Here, we measured several variables (topographical, physical and environmental) to characterize Tahiti petrel nesting habitats and burrows (i.e., width, height, depth and type: rocky cavity, dug into the soil or under a root) on Nemou Island in New Caledonia. The data were clustered using the HCPC (Hierarchical Clustering on Principal Component) method to identify principal habitat groups. This method was combined with logistic regressions to examine the influence of the variables on nest-site selection and breeding success. Our results showed that nest-site selection is linked to habitat groups (a combination of substrate and vegetation data), slope, orientation and soil depth, while breeding success is only influenced by nest characteristics (i.e., burrow type and width). Tahiti petrels prefer to nest on steep slopes in mature forests with rocky substrate and deep soil. Burrows were scatterred in small sub-colonies or isolated pairs, suggesting that nest-site selection depends on habitat quality rather than conspecific density. The study also revealed that breeding success is lower in rocky cavities and increases in burrows with wide entrances. Our nest-site selection survey is the first for the genus *Pseudobulweria*, and provides critical information for designing effective conservation programs in New Caledonia and the Pacific.

## Introduction

Many seabird populations are declining due to invasive alien species (cats *Felix catus*, rats *Rattus spp*. and pigs *Sus scrofa*), fisheries bycatch and various anthropogenic pressures (e.g., nesting habitat destruction, light and noise pollution, egg collection, disturbance and climate change) [1–3]. Over the past 60 years, these threats have contributed to an overall decline of 70% in the global seabird population [3]. Seabirds are considered bio-indicators of marine environmental health [4] and ecosystem engineers on land [5]; therefore, limiting this decline is essential to preserve their natural habitats. On land, conservation and restoration programs, including predator control and nesting habitat improvement, can reduce this decline by expanding existing colonies or restoring historical populations [6]. However, basic knowledge about the biology, ecology and nesting habitat selection (i.e., nest-site selection) of the species is often limited [7–9].

Johnson [10] ranked habitat selection into four category: selection of the physical or geographical range of a species (*first-order selection*), selection of a home range of individual or social group (*second-order selection*), selection of various habitat patches within the home range (*third-order selection*), and selection of specific resources within a habitat patch (*fourth-order of selection*) [11]. Nest-site selection corresponds to the fourth-order selection, in which habitat characteristics are used as specific resources. Studies based on habitat characteristics generally examine nesting site preference by comparing the sites used with available sites [11, 12]. Thus, nest-site selection relies on several factors, including local and micro-climate [13], conspecific social attraction [14, 15], vegetation and topography (i.e., soil type, slope, aspect, elevation) [16–18] and predation [19]. Most seabirds are faithful to their breeding site [20], providing fitness advantages over the years [21]. Thus, understanding the mechanisms of nest-site selection in declining species is essential to plan adapted conservation strategies.

Monitoring breeding success is critical for determining the status of populations and their responses to anthropogenic and environmental stressors. The selection of good-quality nest-sites is a significant predictor of breeding success [22, 23], along with the quality of breeders based on their breeding experience [24]. Besides, for burrow-nesting seabird species like petrels (i.e., order Procellariidae), burrow characteristics including depth, orientation and sinuosity impact breeding success by modulating the birds’ exposure to predation, flooding, high temperature or collapse [17, 23]. However, the relative contribution of nest-site selection and burrow characteristics to breeding success is still understudied in seabirds [25–27].

Previous studies have described the nest-site selection of some burrow-nesting Procellariidae species [18, 28–32], but quantitative information for the genus *Pseudobulweria*, the world’s most threatened seabird genus, remains scarce [33]. *Pseudobulweria* includes one extinct species *(Pseudobulweria rupinarum)* and four extant species. The Beck’s petrel (*Pseudobulweria becki)*, the Fiji petrel (*Pseudobulweria macgillivrayi*) and the Mascarene petrel (*Pseudobulweria aterrima*) are all considered Critically Endangered by the IUCN. The last species, the Tahiti petrel (*Pseudobulweria rostrata*), is a cryptic species currently listed as Near-Threatened, under suspected decline and in lack of quantitative data [34]. The Tahiti petrel faces two major threats: predation by introduced vertebrates (mainly cats and rats) at their breeding sites and destruction of the breeding sites by mining activity [1, 35, 36]. Minor other threats often occur, such as light pollution induced by mining activity and cities, plastic ingestion and bycatch at sea [2, 37]. Its world population is estimated between 10,000 and 20,000 breeding individuals [34, 38], with colonies distributed over several tropical Pacific islands: American Samoa, Fiji, French Polynesia and New Caledonia, for a estimated terrestrial area about 6 850 000 km^2^. However, the exact area for occupancy is not known [34]. The current lack of basic knowledge about the biology, ecology and population distribution of the Tahiti petrel prevents the implementation of efficient conservation or restoration strategies that could reverse population decline. Villard et al. (2006) and Rauzon & Rudd (2014) pioneered the monitoring of Tahiti petrel and provided preliminary data about the vocal repertoire and the breeding biology of the species [39, 40]. Nest site management and threat mitigation actions dedicated to Tahiti petrel are currently confronted with an urgent need for quantitative assessments of its population distribution, nest-site selection, and burrow characteristics [6, 41].

We investigated the Tahiti petrel nest-site selection, burrow characteristics and their influence on breeding success at a New Caledonian breeding colony. Our study specifically aimed to determine: (i) the habitat characteristics that influence the location of the Tahiti petrel nesting sites, and (ii) the main burrow features influencing Tahiti petrel breeding success. We monitored and surveyed Tahiti petrel for the first time in a colony isolated from the most direct human pressures present on the mainland. The set of habitats naturally available on Nemou Island represents the diversity of breeding habitats present in New Caledonia. The Tahiti petrel is a nocturnal and cryptic species. The data collected will help conservation managers identify the most suitable habitats to preserve the Tahiti petrel population, especially before starting mining activities at a new site. This study also intends to improve the general species knowledge.

## Materials and methods

### Ethics statement

This study was conducted with the approval and authorization of the legal authority in charge of the environment and biodiversity of the South Province of New Caledonia: Direction de l’Environnement de la Province Sud, permit n° 836-2019/ARR/DENV. The Tahiti petrel is a legally protected species under the environmental legislation of the South Province. No contact or handling of animals was made during the study.

### Study site and species

**Fig 1:**
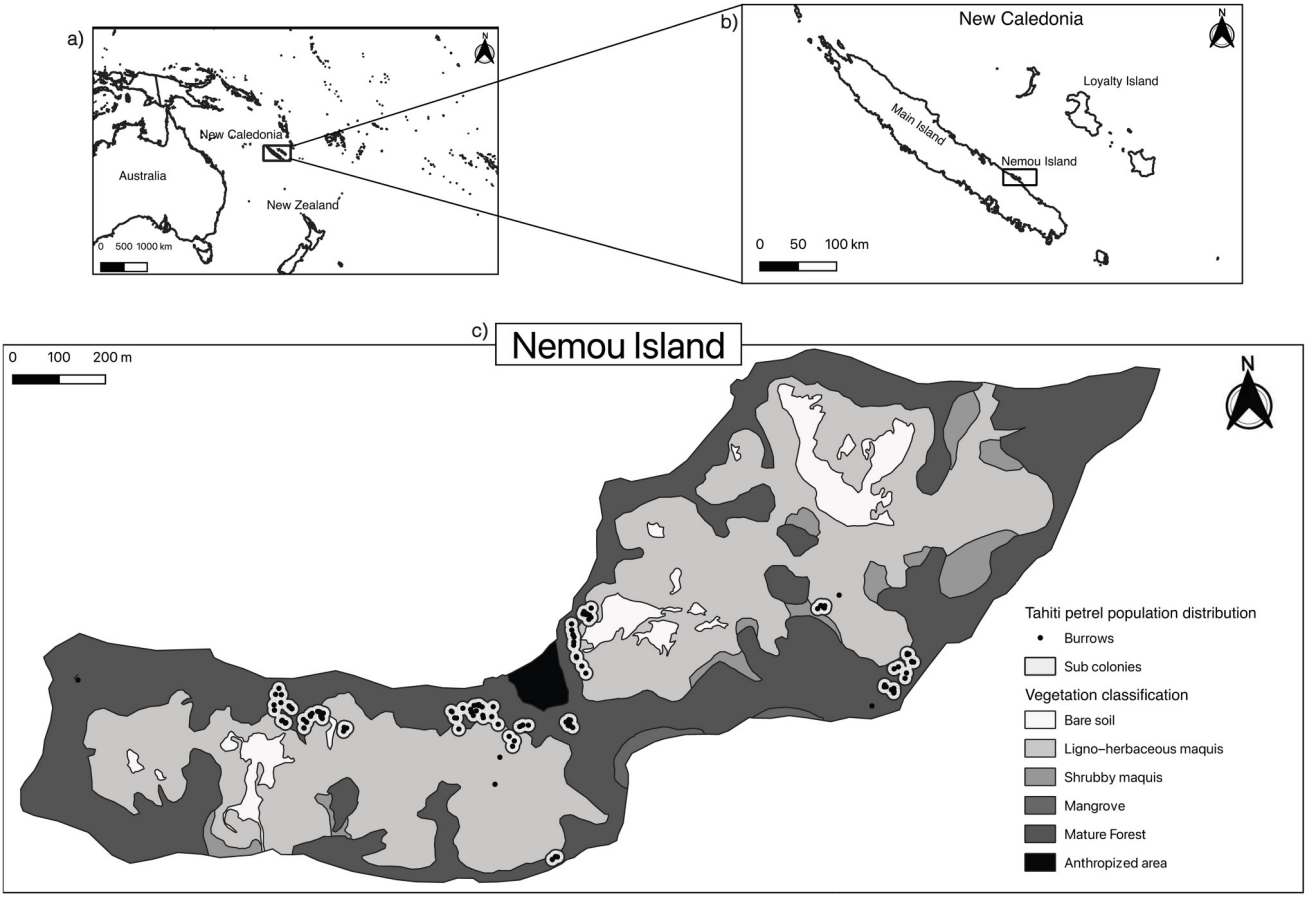
Map of Nemou Island. Location of the island in the Pacific region (a) and New Caledonia (b), and location of Tahiti petrel burrows (n = 116), sub colonies (n = 13) and vegetation classification (c). The anthropized area is where some of the vegetation has been cut down to allow visitors to come ashore and camp in an articficial structure. Republished from the New Caledonia Government and South Province under a CC BY license, with permission from Déborah David and Justin Pilotaz respectively, original copyright 2011.

This study was carried out on Nemou Island (20.38°S, 164.04°E South-West Pacific Fig 1a), located off the eastern coast of New Caledonia (1 km from Grande Terre) (Fig 1b), between May 2018 and July 2020. Nemou is an island of 125 hectares (Fig 1c), rising to 168 m above sea level. It is uninhabited but occasionally visited for the day or camping activities. Invasive species present on the island include rats (*Rattus rattus and Rattus exulans*) and Rusa deers (*Cervus timorensis russa*). A feasibility study for rat eradication showed low rat population abundance (catch rate approximatively 1.5% for a total of 556 trap nights [42]). Before this study, the Tahiti petrel population on the island was estimated to be a few tens of breeding pairs. No other Procellariform species are known to nest on this island. Nemou Island is under the customary authority of Petit Borendi tribe. Customary formalities were carried out with Radji Kainda, the island owner, to gain access to the study site. Radji Kainda himself organized transportation to the study site.

The species is known to breed in two types of habitats: most nest-sites are located in mountainous areas (up to 1,200 m) or high rocky islands (between 40 m and 200 m), while only a few burrows are found in backshore sandy areas. Besides, on mountainous areas, Tahiti petrel nest in forests and maquis [36] but not in the rainforest (personal observations). They also nest on some islets in the lagoon [39]. Like many procellariid species, Tahiti petrels spend most of their time foraging at sea, except during their breeding period, when they depend on terrestrial environments to reproduce. New Caledonian populations breed asynchronously (i.e., breeding cycles extend throughout the year) [43], an atypical feature in Procellariidae [38]. Villard et al. [39] estimated incubation and chick rearing periods to last 55 and 120 days, respectively [39]. Individuals are considered philopatric, with high nest fidelity, as they return to the same breeding site each year and breed in the same burrow for several years [39, 44].

### Nest-site selection

Systematic ground surveys, searching for and mapping Tahiti petrel burrows, were conducted for the first time at Nemou Island throughout 2018. Adult Tahiti petrels return on land at night only to breed, incubate the egg or feed the chick. They display high vocal activity at the beginning of the night. Nocturnal vocal activity monitoring preceded each survey at the beginning of the night when vocal activity peaks (i.e., between 6 pm and 9 pm) [36]. Two observers climbed the two highest ridges of the island (western ridge and eastern ridge of Nemou, Fig 1) to do call counts and listen to the Tahiti petrel activity during the first few hours of 10 nights (see S1 Fig). Positioned simultaneously on each ridge, the observers were able to identify areas of highest vocalization activity over the whole island (> 100 calls / 5 min). These areas were then visually searched to map the breeding burrows. While this approach allowed for mapping all petrel sub colonies, remote and isolated burrows may have been missed.

We recorded the coordinates of each burrow using a GPS (Garmin^®^ GPS 64 S, mean accuracy: ± 3 m). After producing the burrows distribution map from field prospections, we recorded Tahiti petrel nest-site characteristics through a set of biotic and abiotic variables measured in 102 quadrats (8 m x 8 m) [28]. Quadrats were randomly positioned using the searching function for random locations in a polygon in Qgis (3.2, v3.2.3-Bonn, gaussian distribution) so that only half of them included Tahiti petrel nesting areas (n = 51), and all habitats on the island were represented (S1 Fig). Burrow patches in Fig 1 represent a circular buffer of 15 m radius around the GPS locations recorded on top of monitored burrows. Each patch encompasses closely related burrows and surrounding landing/take-off areas. Within each quadrat, 14 topographical, physical and environmental variables were collected [28] (Table 1). Physical variables were measured at the centre of each quadrat, while substrate and vegetation variables were visually estimated (in percent) over the whole surface of each quadrat. We established and oriented all quadrats (North-South and East-West) using a 30 m tape measure and a compass. Elevation, orientation and slope were determined at the center of each quadrat using a GPS (GpsMap 64s), with a hand-held compass and an inclinometer. Because orientation is a circular variable, the mean orientation was calculated using the function *mean*.*circular* of the R package “circular” v.0.4–93 [45, 46]. Soil depth was calculated as the average of the maximum penetration of a metal bar into the soil based on 5 measurements: 4 measurements at 1 m from each corner towards the centre of the plot, and one at the centre [47]. The metal bar was pushed into the ground as far as possible until it encountered an obstruction or was buried. Distance to the coastline was determined using Qgis for each quadrat, and trunk (i.e., the main stem of a living tree) diameter was assessed using a 30 m tape measure at 1 m above the ground.

**Table 1:** Variables measured in 102 quadrats (51 within and 51 outside nesting areas) and around 87 burrows (48 successful and 65 unsuccessful).

	Variables	Units	Nest site selection (14 variables)				Breeding success (16 variables)	
				Quadrats (mean ± se)			Burrows (mean ± se)	
				Within nesting areas	Outside nesting areas		Successful	Unsuccessful
**Physical**	*orientation*	degree	**x**	168.6 ± 19.4	158.4 ± 14.7	**x**	216.6 ± 19.8	165.7 ± 17.6
	*elevation*	meter	**x**	47.5± 4.8	55.4 ± 5.3	**x**	43.5 ± 5.0	49.4 ± 4.9
	*slope*	degree	**x**	33.1 ± 1.4	25.3 ± 1.5	**x**	16.5 ± 1.6	17.9 ± 1.5
	*distance to coastline*	meter	**x**	94.0 ± 8.6	107.4 ± 11.1			
**Substrate**	*outcrop cover*	percent	**x**	13.6 ± 2.8	3.4 ± 1.0			
	*block cover*	percent	**x**	16.0 ± 2.4	12.1 ± 2.4	**x**	19.7 ± 4.3	28.6 ± 4.2
	*scree cover*	percent	**x**	23.5 ± 2.9	27.8 ± 3.3	**x**	15.0 ± 2.3	14.3 ± 2.4
	*loose soil*	percent	**x**	47.2 ± 4.1	60.0 ± 4.6	**x**	65.0 ± 4.1	58.5 ± 4.2
	*soil depth*	cm	**x**	24.3 ± 1.8	18.0 ± 2.2			
**Vegetation**	*tree cover*	percent	**x**	83.9 ± 4.3	68.6 ± 5.2	**x**	75.3 ± 5.1	78.3 ± 4.6
	*shrub cover*	percent	**x**	31.7 ± 2.9	45.0 ± 4.4	**x**	34.8 ± 3.5	38.3 ± 4.0
	*grass cover*	percent	**x**	41.9 ± 4.5	25.0 ± 2.3	**x**	27.1 ± 3.0	25.8 ± 2.5
	*trunk count (diam > 20cm)*	–	**x**	41.5 ± 6.7	27.2 ± 3.0			
	*trunk count (diam < 20cm)*	–	**x**	2.6 ± 0.3	2.3 ± 0.4			
**Burrow**	*entrance width*	cm				**x**	19.5 ± 0.9	22.0 ± 1.0
	*entrance height*	cm				**x**	13.7 ± 1.4	12.5 ± 0.7
	*burrow depth*	cm				**x**	105.3 ± 6.2	119.7 ± 5.3
	*tunnel shape*	no, straight, winding				**x**	–	–
	*entrance protection*	without, with				**x**	–	–
	*type*	root, boulder (BB), soil (BS), rocky (C)				**x**	–	–
**Conspecifics**	*burrow density (3m radius)*	–				**x**	0.5 ± 0.1	0.5 ± 0.1

x indicates variables considered predictors in nesting habitat selection and/or breeding success models of the Tahiti petrel.

### Breeding success

Burrows were monitored every two months between May 2018 and July 2020 (14 field sessions of 5 consecutive days each) to account for breeding asynchrony. We used a "burrowscope", i.e., a miniature endoscopic camera (Adult Tortoise Camera System) connected with virtual reality glasses (Fatshark Dominator V3) and LCD screen (fat Screen 7" diagonal display LCD colour monitor). Each burrow was monitored (empty, eggs, chicks, adults). Only the burrows actively used by Tahiti petrels for breeding (i.e., burrows with eggs, chicks, adults) were included in the analyses. Active breeding burrows were considered successful or unsuccessful based on whether the egg laid led to a fledgling or not. Breeding success was determined by the percentage of eggs that gave a fledgling.

We used 16 variables describing the burrows and their environment to account for burrow characteristics as potential predictors of breeding success (Table 1). Physical, substrate and vegetation variables were measured within a 1 m radius of each burrow. Burrow dimensions were measured using a 30 m tape measure, except for the depth of the burrow, which was obtained by measuring the length of the burrowscope tube that could be inserted into the burrow. Roots and/or stones at the burrow entrance were considered as burrow entrance protection from predators (e.g., rats). Tahiti petrels used three types of burrows: 1) dug under a root or a boulder (BB), 2) dug in the soil (BS), and 3) a rocky cavity that they did not need to dig (C).

### Statistical analyses

All statistical analyses were conducted using the R software version 4.0.3 [48]. We replicated the same analytical framework on two datasets to determine the best predictors of (i) nest-site selection and (ii) breeding success of Tahiti petrels. The binary response variable of the first dataset was presence (1) and absence (0) of burrows for each of the 102 quadrats, so the unit of replication in the model is the quadrat. The second dataset consisted of 87 active burrows monitored throughout the study (May 2018 to July 2020). With this second dataset, we considered breeding success as a binary response variable (i.e., success (1), failure (0)), so the burrow is the unit of replication of the model. Variations along the North-South and East-West axes were included in the models using the cosinus and sinus of the orientation angle) [46]. Multicollinearity among explanatory variables was assessed using the function *cor* of the R package Stats v.4.0.3 [48]. Variables correlated beyond 50% (e.g., substrate and vegetation cover variables) were considered highly correlated with a risk of multicollinearity. Because substrate and vegetation cover variables were highly correlated, the following three-step analysis was designed: 1) a Hierarchical Clustering on Principal Component (HCPC) to cluster the multiple and non-independent habitat predictors, 2) a logistic regression including clusters assignments as categorical variables with the other available predictors and, 3) a downward stepAIC to select the best of all available and non-independent predictors [49, 50].

In step 1, an HCPC method was applied to reduce the number of vegetation and substrate cover variables to a smaller set of uncorrelated components using the "FactoMiner" v.2.3 and "Factoextra" v.1.0.7 R packages [51, 52]. The number of clusters was chosen based on the “FactoMiner” package’s suggested optimal level. Using Ward’s minimum variance criterion, the HCPC method clusters variables into groups based on the distance (i.e., inherent similarity) between them to minimize the total within-cluster variance. As a result, the vegetation and substrate cover variables were merged into 3 clusters for the two analyses, depending on their position along the two dimensions of a Principal Component Analysis (PCA). The first two components of the PCA extract the most important information from the dataset. The main variables associated with the PCA are indicated with their contribution (i.e., c). The PCA function scaled and normalized each variable with a zero mean and a unit standard deviation. Thus, using HCPC to develop a typology based on the first two components of the PCA provides a more consistent and accurate classification because noise is reduced in the analysis. For each cluster, three values represented the contributions of the variables: the Overall Mean of the variable (OM), the Mean value In the Cluster (MIC) and a value-test (v.test) representing the sign and significance of the MIC-OM difference. A v.test value greater than 1.96 indicates a significant contribution of the variable to the cluster at a 95% confidence level [53].

In step 2, we modelled nest-site selection with the physical variables measured in the quadrats (i.e., orientation, slope, elevation and distance to the coastline, Table 1) and the HCPC habitat clusters using logistic regression. We then modelled breeding success using burrow characteristics (Table 1), density, orientation, elevation, slope and HCPC habitat clusters using logistic regression as well.

In step 3, after constructing full logistic regressions models, we conducted a downward stepwise predictors selection based on corrected Akaike’s Information Criteria for limited sample sizes (AICc) using the R package "MASS" v.7.3–53 [53, 54]. This method determines which explicative variable removal would result in the most significant AICc reduction beginning with the full model for each response variable. These explicative variables were then removed, and the procedure repeated until no AICc reduction was possible. Models with the lowest AICc were ranked with the *aictab* function of the R package "AICmodavg" v 2.3–1 [55], using Akaike’s Information Criterion for limited sample sizes (AICc), the difference between the model AICc and the lowest AICc (ΔAICc) and AICc weights (wAICc). Models with ΔAICc ≤ 2 and 95% confidence intervals not overlapping zero were selected [56] (see S1 and S2 Tables). Models with ΔAICc ≤ 2 were then averaged with the "MuMin" R package v.1.43.14 [56, 57]. To avoid any overestimation of averaged estimates, we provide estimates from the full-model averaging, meaning that parameters estimates were set to 0 when not included in a model [58].

We built and tested our logistic regressions, the best model for binary response variables, using the R package "stats" v.4.0.3 [48]. Model assumptions and predictive performances were checked statistically [59, 60]. The overdispersion index validated the absence of overdispersion in our data. Odds ratios represent the ratio of the probability of the event of interest occuring (i.e., burrow presence (1) or success (1)) to the probability of it not occurring. The pseudo R^2^ estimated the predictive performance of our models [61, 62]. Values are shown with a mean ± standard error.

## Results

### Burrow distribution and nest-site selection

A total of 116 burrows were found across the whole island. Among these burrows, 113 showed evidence of occupation by Tahiti petrels (i.e., feather, fresh guano), of which 87 showed evidence of breeding (i.e., egg, chick or adult). Burrows were relatively distant from each other, with a burrow density of 0.03 ± 0.01 per m^2^ (i.e., within quadrats) (Table 1) and an average distance to the nearest burrow of 7.4 ± 0.8 m. The study detected 13 sub-colonies of 9 ± 1.6 burrows, and five isolated burrows, totalising 87 breeding pairs (Fig 1).

**Fig 2:**
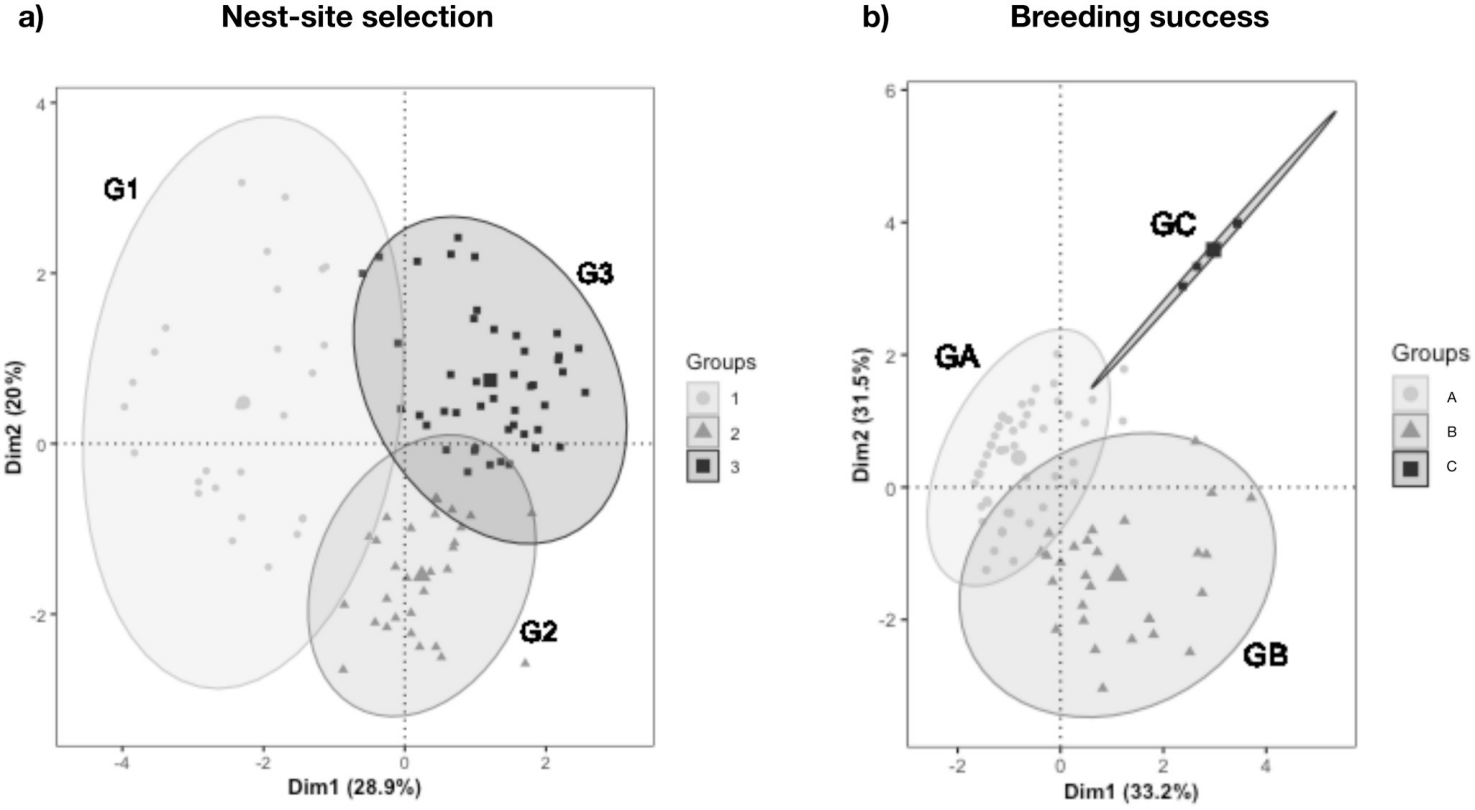
Classification of habitat groups based on Principal Component Analysis (PCA) and Hierarchical Clustering on Principal Components (HCPC) (i.e., dimensions) applied to substrate and vegetation data in (a) the 102 quadrats characterized for the analysis of Tahiti petrel nest-site selection and (b) the 87 1-m radius plots around burrows used to identify factors influencing Tahiti petrel breeding success. Axes represent scores for every 102 quadrats and 87 1-m radius plots from variables included in the PCA. The percentage in brackets is the variation of the variables explained by each dimension. Plots were classified into three groups for both the Tahiti petrel nest-site selection and breeding success. Each group represented a specific habitat type, as described in the results section. For nesting habitat selection (a): G1 = open habitats, G2 = closed habitats and G3 = rocky habitats. For breeding success (b): GA = habitat mainly with tree and scree, GB = habitat mainly with rocky substrate and GC = habitat with low vegetation.

**Fig 3:**
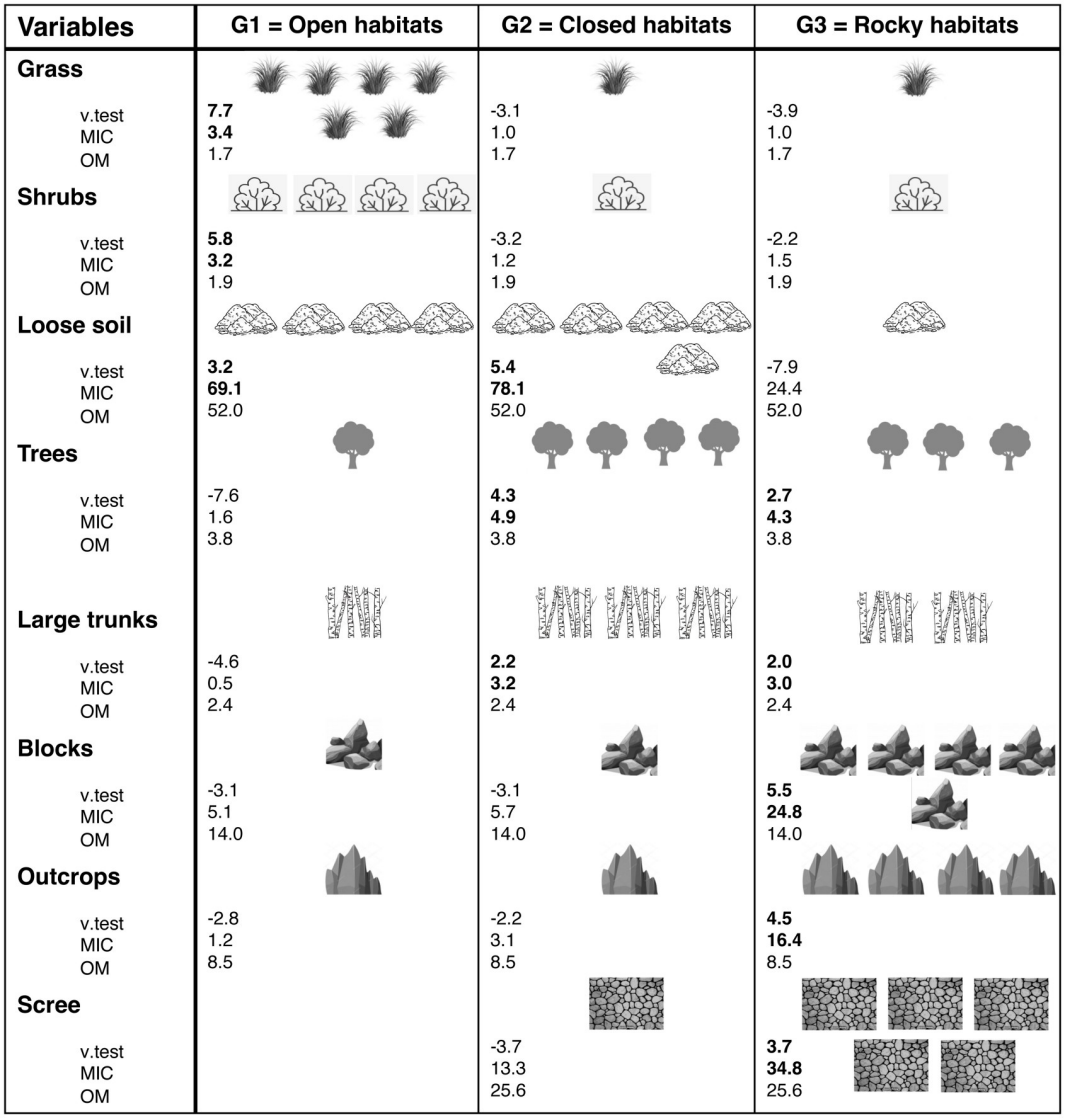
V.test value (positive or negative), mean in category value (MIC) and overall mean value (OM) of habitat selection groups by Tahiti petrels, based on Hierarchical Clustering on Principal Components (HCPC). Group 1 (G1): habitat mainly composed of grass, shrubs and loose soil; Group 2 (G2): habitat mainly composed of loose soil, trees and large trunks; Group 3 (G3): habitat mainly composed of blocks, outcrops, scree and trees with large trunks.

The first two principal components given by the PCA performed on vegetation and substrate cover variables explained 28.9% and 20.0% of their total variation, respectively. The first principal component was mainly associated with grass (c = 24.2%), tree (*c* = 18.5%) and shrub (c = 15.7%) cover. The second principal component was mainly linked to loose soil (c = 35.8%), scree (c = 18.6%) and tree (c = 13.5%) cover (Fig 2a). The HCPC analysis classified vegetation and substrate variables into three "habitat" groups (Fig 3). Group 1 (G1) represented “open” habitats, characterized by loose soil (69%), grassy (mean in category (MIC) = 3.4) and shrubby vegetation (MIC = 3.2). In contrast, group 2 (G2) represented “closed” habitats with mature forest (Tree: MIC = 4.9; Trunks: MIC = 3.2) and 78% of loose soil. The last group (G3) represented "rocky" habitats with rocks (blocks, outcrops and scree) and trees (MIC = 4.3) (Fig 3).

The best model describing nest-site selection by Tahiti petrels for nesting included habitat groups (as defined by the HCPC analysis), slope, orientation and soil depth (ΔAICc = 0.0, wAICc = 0.63, CI = 5.03–12.64, S1 Table). Rocky and closed habitats were the best predictors of burrow presence, with rocky habitats much more likely to have burrows (Table 2a). Steep slopes were the model’s second major predictor, with a positive relationship between slope and Tahiti petrel burrow presence (Table 2a). North and east orientation also had a positive effect. Finally, Tahiti petrel burrow presence was positively influenced by soil depth (Table 2a). The model explained 42% of the Tahiti petrel nest-site selection variation (R^2^ = 0.42).

**Table 2:** Summary tables of logistic regressions explaining the influence of nest-site selection on burrow presence (a) and the factors influencing breeding success (b) of the Tahiti petrel at Nemou Island.

	Estimate	Std.error	*P*—value		*Odds Ratios*
**a) Nest-site selection variables**					
Intercept	-7.76	1.98	1.08e-04	***	–
Cos Orientation (North-South)	1.15	0.37	0.002	**	3.2
Sin Orientation (East-West)	1.36	0.57	0.02	*	4.2
Slope	0.09	0.03	0.007	**	1.1
G2	3.42	1.13	0.003	**	42.4
G3	3.85	1.08	0.0004	***	58.4
Soil depth	0.07	0.02	0.004	**	1.1
Distance to the nearest coastline	0.01	0.01	0.07	.	–
**b) Breeding success variables**					
Intercept	-2.22	1.35	0.10		–
Burrow entrance width	0.10	0.04	0.02	*	1.1
Burrow depth	0.01	0.01	0.08	.	1.0
Burrow Type BB	-0.36	0.52	0.48	.	–
Burrow Type C	-1.75	0.71	0.01	*	0.7

Asterisks indicate significant difference as follows:

* p <0.05,

** p < 0.01,

*** p < 0.001.

### Burrow characteristics and factors influencing breeding success

Burrow entrances were wide (about 20.8 ± 0.6 cm) and located on steep slopes, 55.4% (i.e., 17.2° ± 1.1°) on average across the entire elevation gradient (Table 1). Breeding success was 0.32 ± 17.5 on average for the 87 breeding attempts monitored throughout the 26 months of our study. The first two principal components given by the PCA performed on vegetation and substrate cover variables explained 33.2% and 31.5% of their total variation, respectively. The first principal component was mostly correlated to trees (c = 32.5%), grass (c = 21.8%) and blocks (c = 15.9%). The second principal component was mainly linked to loose soil (c = 41.2%), blocks (c = 33.5%) and grass (c = 14.1%) (Fig 2b). The final typology of the HCPC results also classified vegetation and substrate variables into three groups, but none was significant in the final model. Group A was mainly composed of trees (MIC = 4.3), loose soil and scree (73.7% and 20.0% cover, respectively). Group B was mainly composed of a rocky substrate (60% of block cover). In contrast, the last group was made of low vegetation, including grass (MIC = 4.7), shrubs (MIC = 3.7) and loose soil.

The best model included burrow type, width and depth as predictors of Tahiti petrel breeding success (ΔAICc = 0.00, wAICc = 0.50, CI = 0.3–5.39, S2 Table). Burrow type was the first predictor of the model and showed a negative effect of rocky cavities (C) on breeding success (Table 2b), but there was no significant relationship between burrows dug under blocks or roots (BB) and breeding success. Burrow entrance width was the second determinant of the model, with a positive relationship between burrow width and breeding success. Burrow depth had a slight, yet insignificant, positive impact on breeding success (Table 2b). However, this model explained only 12% of Tahiti petrel breeding success variation (R^2^ = 0.12).

## Discussion

We provide the first assessment of the most suitable habitats for breeding Tahiti petrels. Mature trees and rocky substrates appear as critical features in the species breeding behaviour. We show that the width of the burrow entrances is positively correlated to breeding success.

### Habitat suitability

Our results show that habitat groups, slope, orientation and soil depth influence the selection of nesting sites by Tahiti petrel. Closed and rocky habitats most significantly explained the occurrence of Tahiti petrel burrows at our study site. These habitats include mature trees (i.e., trees with large trunks) on which Tahiti petrels like to climb to take off [63]. Therefore, a plausible hypothesis is that Tahiti petrels favour closed and rocky habitats for nesting because they offer easy take-off points [18]. Indeed, adult Tahiti petrels can walk up to 50 m away from their burrow to reach a mature tree from which they can take off, and claw marks have been observed on several trunks (Pagenaud, pers. obs.). Similar behaviour was observed for Cook’s petrels, which can walk up to 100 m to take off from a tree [18]. Mature trees could also represent a landmark for adult breeders to locate their burrows. Mature forests are often preferred because they provide shelter from intense weather, sun or aerial predators [64] and a more stable substrate thanks to well-developed root systems. Conversely, Tahiti petrels seem to avoid areas without branches, and only one burrow (out of 113) was found in such a situation. Tahiti petrels also prefer rocky habitats for nesting, especially with scree and blocks. This type of substrate may improve the stability of the cavity [47, 65] and minimize burrow collapse due to heavy rainfall or large mammals (i.e., rusa deer on Nemou Island) [65]. Moreover, outcrops can be used as take-off platforms like trees.

Our nest-site selection models revealed that Tahiti petrels prefer northerly or easterly orientated slopes (i.e., facing the prevailing north-east winds), perhaps because they facilitate landing through drag force and take-off through lift force [44]. Finally, most Tahiti petrel burrows were found on steep slopes, a characteristic often observed in other seabirds, particularly Procellariiformes [19, 64–66]. Steep slopes generally provide better water drainage, preventing water from entering the burrow [67]. Slopes also facilitate burrow excavation [68] and ease bird take-off [65, 69]. We also found a link between soil depth and the presence of Tahiti petrel burrows. Burrowing seabirds usually select soft or deep soil, which is easier to dig in [28, 70, 71].

We recommend that this set of predictors (i.e., habitat groups, slope, orientation and soil depth) be considered in habitat modelling analyses to identify other potential breeding sites. Applications include the identification of suitable nest-sites for Tahiti petrels in mining impacted areas on Grande Terre.

In addition, the overall distribution of Tahiti petrels may be influenced by the availability of breeding sites (mature forest, rocky habitat, steep slope, soil depth), which can be sought out in other archipelagos (e.g., Fiji, American Samoa, French Polynesia). Despite Nemou’s small surface area and generally low altitude, the island hosts a diverse range of habitats, which match the majority of available known habitats for Tahiti petrels (as previously cited). Thus, the diversity of habitats on the island has been assumed as representative of habitats on Grande Terre and of petrel habitats in general in the region.

### Determinants of breeding success

Breeding success was lower in rocky cavities but improved when burrow entrances were wider. However, the environment directly surrounding the burrows seems to have no influence on breeding success compared to burrow features. Surprisingly, wider burrow entrances enhance Tahiti petrel breeding success. Indeed, smaller burrow entrances generally limit access to predators and protect from weather conditions [22, 72]. However, regular monitoring of burrows revealed three dead Tahiti petrel fledgling chicks trapped in burrow entrances smaller than average (about 10 cm wide). Larger burrow entrances could then be easier to pass through, with less risk of chicks getting trapped when they come out to exercise their wings daily or to fledge. A wider entrance could also make it easier for adults’ access to the nest chamber [73]. Besides, a wide entrance enhances burrow ventilation and thermoregulation, improving chick survival [74].

As confirmed by previous observations, substrate type can significantly affect breeding success in seabirds [75]. Tahiti petrels prefer digging their burrows near rocky substrates, increasing roof stability during and after building the burrow. However, natural rocky cavities or crevices, where the nest chamber is made of stones or rocks (i.e., not dug by Tahiti petrels), could increase the probability of egg breaking. This feature has been reported for other procellariiform species such as the Macaronesian shearwater (*Puffinus lherminieri baroli*) or Cory’s shearwater (*Calonectris diomedea*) [16, 76].

Overall, this study provides the first data on the breeding success rate for the species. This breeding success rate was relatively low (32%) compared to other Procellariids which typically have breeding success rates near or above 50% [77–81]. Breeding success can be affected at any stage (i.e., egg, chick or fledgling) by invasive predators such as rats, pigs or feral cats [82, 83]. However, the limited abundance of invasive predators on Nemou Island is unlikely to impact Tahiti petrel breeding success. A camera trap survey could provide additional data on the potential effects of rats or other nocturnal predators on the Tahiti petrel demography. Nest desertion during bad weather could also apply (e.g., storms) [84].

Further studies on the breeding biology of the species are needed to enhance our understanding of these interactions and identify potential additional factors impacting breeding success. The predictive performance of our breeding success model could be improved by including other predictors of the Tahiti petrels’ breeder quality. Indeed, age, body size, foraging performance, experience and intra-specific competition could directly affect hatching success, fledging success and breeding success [85–87].

Finally, long-term monitoring of Tahiti petrel breeding success would provide additional data on the interannual variability of hatching, fledging and breeding success and could help identify the factors driving this variability [88, 89].

### Tahiti petrel population distribution

Our results indicate that Tahiti petrel burrows are isolated from each other with a burrow density of only 0.03 ± 0.01 per m^2^. These results suggest that the species prefers to nest in low density within a large area. In contrast, most burrowing Procellariidae nest in crowded colonies like the wedge-tailed shearwater or Cory’s shearwater [16, 90, 91]. Also, Tahiti petrels tend to nest in small sub-colonies or isolated pairs (i.e., one burrow). This patchy distribution suggests that nest-site selection is based on habitat quality rather than conspecific density [92]. Consequently, preserving nesting habitat is a critical factor for conservation.

In addition, this uneven distribution means that searching for burrows in large areas requires more effort and experience. This information can help observers to improve breeding ground survey strategies: they can identify smaller evidence (e.g., feather, down, guano) rather than searching for a colony.

Out of all the sites identified in New Caledonia (n = 34), our survey revealed that Nemou Island was the only accessible site where a large breeding population of Tahiti petrel (between 100 and 200 pairs) can be studied. This population of Tahiti petrels is the largest recorded to date in New Caledonia.

### Implications for conservation and future research

We believe that Nemou Island should be considered a refuge for Tahiti petrels in New Caledonia. The island is free from mining activities or other Procellariform species (e.g., the wedge-tailed shearwater *Ardenna pacifica*) that could compete with nesting sites and burrows. It is also very little impacted by human activities or introduced rodents and is free from feral cats. Therefore, Nemou Island is a valuable study site for conserving this poorly known, rare and declining species. Further actions should include raising public awareness of the species, increasing protection of nesting habitats, and eradicating rodents from Nemou Island.

Our study provides the first data about the Tahiti petrel nest-site selection and burrow characteristics. These data are essential for conservation purposes and can be used to (i) develop predictive habitat models that identify and map potential nesting areas in New Caledonia or other islands in the Pacific, (ii) facilitate the search for nesting sites in the vast mountain range of the main island of New Caledonia, (iii) and improve population recovery [93, 94].

## Supporting information

S1 Fig Map of the quadrats distribution on Nemou Island. The map shows (i) the position of quadrats whitin and outisde Tahiti petrel (TP) nesting habitat, (ii) the location of the edge of the TP sub colonies, and (iii) the vegetation classification. Quadrats are shown to scale. The black section (Anthropized area) is the area where vegetation was cut out to set up a camping area.
(PDF)

S1 Table Predictive habitat models selection for Tahiti petrel based upon corrected Akaike’s Information Criterion. (PDF)

S2 Table Models statistics used to predict the influence of burrow characteristics and environmental variables on the Tahiti petrel breeding success. (PDF)

## References

[1] Croxall JP, Butchart SHM, Lascelles B, Stattersfield AJ, Sullivan B, Symes A, et al. Seabird conservation status, threats and priority actions: a global assessment. Bird Conserv Int. 2012; 22: 1–34. doi: 10.1017/S0959270912000020

[2] Dias MP, Martin R, Pearmain EJ, Burfield IJ, Small C, Phillips RA, et al. Threats to seabirds: A global assessment. Biol Conserv. 2019; 237: 525–537. doi: 10.1016/j.biocon.2019.06.033

[3] Paleczny M, Hammill E, Karpouzi V, Pauly D. Population trend of the world’s monitored seabirds, 1950–2010. PLoS One. 2015; 10: e0129342. doi: 10.1371/journal.pone.0129342 26058068 PMC4461279

[4] Parsons M, Mitchell I, Butler A, Ratcliffe N, Frederiksen M, Foster S, et al. Seabirds as indicators of the marine environment. ICES J Mar Sci. 2008; 65: 1520–1526. doi: 10.1093/icesjms/fsn155

[5] Smith J, Mulder C, Ellis J. Seabirds as ecosystem engineers: nutirent inputs and physical distrubance. Seabird islands: ecology, invasion, and restoration. Oxford Unversity Press; 2001.

[6] Jones HP, Kress SW. A review of the world’s active seabird restoration projects. J Wildl Manage. 2012; 76: 2–9. doi: 10.1002/jwmg.240

[7] Rodríguez A, Arcos JM, Bretagnolle V, Dias MP, Holmes ND, Louzao M, et al. Future directions in conservation research on petrels and shearwaters. Front Mar Sci. 2019; 6: 1–27. doi: 10.3389/fmars.2019.00094

[8] Probst JM, Le Corre M, Thébaud C. Breeding habitat and conservation priorities in *Pterodroma baraui*, an endangered gadfly petrel of the Mascarene archipelago. Biol Conserv. 2000; 93: 135–138. doi: 10.1016/S0006-3207(99)00114-7

[9] Militão T, Dinis HA, Zango L, Calabuig P, Stefan LM, González-Solís J. Population size, breeding biology and on-land threats of Cape Verde petrel (*Pterodroma feae*) in Fogo Island, Cape Verde. PLoS One. 2017; 12: e0174803. doi: 10.1371/journal.pone.0174803 28369105 PMC5378397

[10] Johnson JB, Omland KS. Model selection in ecology and evolution. Trends Ecol Evol. 2004; 19: 101–108. doi: 10.1016/j.tree.2003.10.013 16701236

[11] Johnson DH. The comparison of usage and availability measurements for evaluating resource preference. Ecolo. 1980; 61: 65–71.

[12] Jones J. Habitat selection studies in avian ecology: A critical review. Auk. 2001; 118: 557–562. doi: 10.2307/4089822

[13] Heenan CB. An overview of the factors influencing the morphology and thermal properties of avian nests. Avian Biol Res. 2013; 6: 104–118. doi: 10.3184/003685013X13614670646299

[14] Buxton RT, Jones IL. An experimental study of social attraction in two species of storm-petrel by acoustic and olfactory cues. Condor. 2012; 114: 733–743. doi: 10.1525/cond.2012.110091

[15] VanderWerf EA, Young LC, Kohley CR, Dalton ME, Fisher R, Fowlke L, et al. Establishing Laysan and black-footed albatross breeding colonies using translocation and social attraction. Glob Ecol Conserv. 2019; 19: e00667. doi: 10.1016/j.gecco.2019.e00667

[16] Ramos JA, Monteiro LR, Sola E, Moniz Z. Characteristics and competition for nest cavities in burrowing Procellariiformes. Condor. 1997; 99: 634–641. doi: 10.2307/1370475

[17] Schumann N, Dann P, Arnould JPY. Use of terrestrial habitats by burrow-nesting seabirds in south-eastern Australia. Emu. 2013; 113: 135–144. doi: 10.1071/MU12088

[18] Rayner MJ, Hauber ME, Clout MN. Breeding habitat of the Cook’s Petrel (*Pterodroma cookii*) on Little Barrier Island (Hauturu): implications for the conservation of a New Zealand endemic. Emu. 2007; 107: 59–68. doi: 10.1071/mu06038

[19] Barros Á, Romero R, Munilla I, Pérez C, Velando A. Behavioural plasticity in nest-site selection of a colonial seabird in response to an invasive carnivore. Biol Invasions. 2016; 18: 3149–3161. doi: 10.1007/s10530-016-1205-3

[20] Bried J, Jouventin P. Site and mate choice in seabirds: an evolutionary approach. Biology of marine birds. 2002. p. 263–305.

[21] Stenhouse IJ, Montevecchi W a. Habitat utilization and breeding success in Leach’s Storm-Petrel: the importance of sociality. Can J Zool. 2000; 78: 1267–1274. doi: 10.1139/z00-065

[22] Michielsen RJ, Ausems AN, Jakubas D, Pętlicki M, Plenzler J, Shamoun-Baranes J, et al. Nest characteristics determine nest microclimate and affect breeding output in an Antarctic seabird, the Wilson’s storm-petrel. PLoS One. 2019; 14: e0217708. doi: 10.1371/journal.pone.0217708 31194763 PMC6564424

[23] Mariné M, Cadiou B. Breeding success, nest site fidelity and mate fidelity in the European Storm-petrel *Hydrobates pelagicus*. Seabird. 2019; 32: 46–58.

[24] Harris MP, Wanless S, Barton TR, Elston DA. Nest site characteristics, duration of use and breeding success in the Guillemot *Uria aalge*. Ibis (Lond 1859). 1997; 139: 468–476. doi: 10.1111/j.1474-919X.1997.tb04660.x

[25] Hamer KC, Schreiber EA, Burger J. Breeding biology, life histories, and life-history-environnement interactions in seabirds. Biology of marine birds. CRC Press; 2001. p. 217–261.

[26] Bourgeois K, Dromzée S, Vidal E. Relationships between nest-cavity and mate selection, reproductive performance and fidelity in the mediterranean endemic yelkouan shearwater *Puffinus yelkouan*. Acta Ornithol. 2014; 49: 9–22. doi: 10.3161/000164514X682850

[27] Holmes G, Koloski L, Nol E. Nest-site selection of a subarctic-breeding shorebird: evidence for tree avoidance without fitness consequences. Can J Zool. 2020; 98: 573–580. doi: 10.1139/cjz-2019-0264

[28] Bourgeois K, Vidal E, Comor V, Legrand J, Dromzee S. Colony-Site Selection Drives Management Priorities for Yelkouan Shearwater Populations. J Wildl Manage. 2008; 72: 1188–1193. doi: 10.2193/2007-052

[29] D’entremont KJN, Zitske LM, Gladwell AJ, Elliott NK, Mauck RA, Ronconi RA. Breeding population decline and associations with nest site use of Leach’s Storm petrels on Kent island, New Bunswick from 2001 to 2018. Avian Conserv Ecol. 2020; 15: 1–11. doi: 10.5751/ACE-01526-150111

[30] Satgé YG, Rupp E, Brown A, Jodice PG. Habitat modelling locates nesting areas of the endangered black-capped petrel *Pterodroma hasitata* on Hispaniola and identifies habitat loss. Bird Conserv Int. 2020; 1–18. doi: 10.1017/S0959270920000490

[31] Troy JR, Holmes ND, Veech JA, Raine AF, Green MC. Habitat suitability modeling for the Newell’s shearwater on Kauai. J Fish Wildl Manag. 2014; 5: 315–329. doi: 10.3996/112013-JFWM-074

[32] Whitehead AL, Lyver POB, Jones CJ, Bellingham PJ, MacLeod CJ, Coleman M, et al. Establishing accurate baseline estimates of breeding populations of a burrowing seabird, the grey-faced petrel (*Pterodroma macroptera gouldi*) in New Zealand. Biol Conserv. 2014; 169: 109–116. doi: 10.1016/j.biocon.2013.11.002

[33] Titmus AJ. Investigating spatiotemporal distribution and habitat use of poorly understood Procellariiform seabirds on a remote island in American Samoa. Doctoral dissertation, University of Hawaii. 2017.

[34] BirdLife International. Species factsheet: *Pseudobulweria rostrata*. Downloaded from http://wwww.birdlife.org on 16 september 2021. 2018.

[35] Palmas P, Jourdan H, Rigault F, Debar L, De Meringo H, Bourguet E, et al. Feral cats threaten the outstanding endemic fauna of the New Caledonia biodiversity hotspot. Biol Conserv. 2017; 214: 250–259. doi: 10.1016/j.biocon.2017.08.003

[36] Pagenaud A, Bourgeois K, Dromzée S, Thibault M, Chagneau G, Barré N, et al. Tahiti Petrel *Pseudobulweria rostrata* population decline at a nickel-mining site: A critical need for adapted conservation strategies. Bird Conserv Int. 2021; 1–13. doi: 10.1017/S0959270921000113

[37] Rodríguez A, Holmes ND, Ryan PG, Wilson KJ, Faulquier L, Murillo Y, et al. Seabird mortality induced by land-based artificial lights. Conserv Biol. 2017; 31: 986–1001. doi: 10.1111/cobi.12900 28151557

[38] Brooke M. Albatrosses and petrels across the world. Oxford University Press; 2004.

[39] Villard P, Dano S, Bretagnolle V. Morphometrics and the breeding biology of the Tahiti Petrel *Pseudobulweria rostrata*. Ibis (Lond 1859). 2006; 148: 285–291. doi: 10.1111/j.1474-919X.2006.00528.x

[40] Rauzon MJ, Rudd AB. Vocal repertoire of the Tahiti Petrel *Pseudobulweria rostrata*: A preliminary assessment. Mar Ornithol. 2014; 42: 143–148.

[41] Miskelly CM, Taylor GA, Gummer H, Williams R. Translocations of eight species of burrow-nesting seabirds (genera *Pterodroma*, *Pelecanoides*, *Pachyptila* and *Puffinus*: Family *Procellariidae*). Biol Conserv. 2009; 142: 1965–1980. doi: 10.1016/j.biocon.2009.03.027

[42] Baudal-Franceschi J. Etude de faisabilité d’une éradication des rats pour la préservation du petrel de Tahiti (*Pseudobulweria rostrata*) sur l’îlot Nemou. 2011.

[43] Ravache A, Bourgeois K, Weimerskirch H, Pagenaud A, de Grissac S, Miller M, et al. Behavioral and trophic segregations help the Tahiti petrel to cope with the abundance of wedge-tailed shearwater when foraging in oligotrophic tropical waters. Sci Rep. 2020; 10: 1–18. doi: 10.1038/s41598-019-56847-4 32934324 PMC7492251

[44] Warham J. The petrels: their ecology and breeding systems. Academic Press. London; 1990.

[45] Lund U, Agostinelli C, Agostinelli MC. Package « circular ». Repos CRAN. 2017. https://www.topsoe.com/processes/sng.

[46] Al-Daffaie K, Khan S. Logistic regression for circular data. AIP Conference Proceedings. AIP Publishing LLC; 2017. p. 030022.

[47] Bourgeois K, Vidal E, Comor V, Legrand J, Dromzee S. Colony-site selection drives management priorities for Yelkouan shearwater populations. J Wildl Manage. 2008; 72: 1188–1193. doi: 10.2193/2007-052

[48] R Development Core Team. R: A language and environment for statistical computing. R Fundation for Statistical Computing, Vienna, Austria; 2016.

[49] Stienen EW, Courtens W, Vanermen N, Verstraete H. Long-term monitoring study of beached seabirds shows that chronic oil pollution in the southern North Sea has almost halted. Mar Pollut Bull. 2017; 115: 194–200. doi: 10.1016/j.marpolbul.2016.12.019 27986298

[50] Camprasse EC, Cherel Y, Arnould JP, Hoskins AJ, Bost CA. Combined bio-logging and stable isotopes reveal individual specialisations in a benthic coastal seabird, the Kerguelen shag. PLoS One. 2017; 12: 1–18. doi: 10.1371/journal.pone.0172278 28264057 PMC5338780

[51] Lê S, Josse J, Husson F. FactoMineR: An R package for multivariate analysis. J Stat Softw. 2008; 25: 1–18. doi: 10.18637/jss.v025.i01

[52] Kassambara A, Mundt F. Package « factoextra ». Extr Vis results Multivar data Anal. 2017; 76. http://www.sthda.com/english/rpkgs/factoextra BugReports.

[53] Venables WN, Ripley B. Random and mixed effects. Modern applied statistics with S. 2002. p. 271–300.

[54] Akaike H. Information theory and an extension of the maximum likelihood principle. Selected papers of Hirotugu Akaike. 1973. p. 199–213.

[55] Mazerolle MJ, Mazerolle MMJ. Package ‘ AICcmodavg ‘. R Packag. 2017; 281.

[56] Burnham K, Anderson D. Model selection and multimodel inference: a practical information-theoretic approach. Springer Science & Business Media; 2002.

[57] Barton K. MuMIn: Multi-model inference. R package version 1.43.17. http://CRAN.R-project.org/package=MuMIn. 2015.

[58] Symonds MRE, Moussalli A. A brief guide to model selection, multimodel inference and model averaging in behavioural ecology using Akaike’s information criterion. Behav Ecol Sociobiol. 2011; 65: 13–21. doi: 10.1007/s00265-010-1037-6

[59] Zuur AF, Ieno EN, Elphick CS. A protocol for data exploration to avoid common statistical problems. Methods Ecol Evol. 2010; 1: 3–14. doi: 10.1111/j.2041-210x.2009.00001.x

[60] Zuur AF, Ieno EN. A protocol for conducting and presenting results of regression-type analyses. Methods Ecol Evol. 2016; 7: 636–645. doi: 10.1111/2041-210X.12577

[61] Nakagawa S, Schielzeth H. A general and simple method for obtaining R^2^ from generalized linear mixed-effects models. Methods Ecol Evol. 2013; 4: 133–142. doi: 10.1111/j.2041-210x.2012.00261.x

[62] Vittinghoff E, McCulloch CE. Relaxing the rule of ten events per variable in logistic and cox regression. Am J Epidemiol. 2007; 165: 710–718. doi: 10.1093/aje/kwk052 17182981

[63] Warham J. The behaviour, population biology and physiology of the petrels. Academic Press; 1996.

[64] Sullivan W, Wilson KJ. Differences in habitat selection between Chatham petrels (*Pterodroma axillaris*) and Broad-billed prions (*Pachyptila vittata*): Implications for management of burrow competition. N Z J Ecol. 2001; 25: 65–69. doi: 10.1071/MU00058

[65] Brandt CA, Parrish JK, Hodges CN. Predictive approaches to habitat quantification: Dark-rumped petrels on Haleakala, Maui. Auk. 1995; 112: 571–579. doi: 10.1093/auk/112.3.571

[66] Buxton RT, Anderson D, Moller H, Jones CJ, Lyver POB. Release of constraints on nest-site selection in burrow-nesting petrels following invasive rat eradication. Biol Invasions. 2015; 17: 1453–1470. doi: 10.1007/s10530-014-0807-x

[67] Stokes D, Boersma PD. Effects of substrate on the distribution of Magellanic Penguin (*Spheniscus magellanicus*) burrows. Auk. 1991; 108: 923–933. doi: 10.1093/auk/108.4.923

[68] Cruz-Delgado F, González JA, Wiedenfeld DA. Breeding biology of the critically endangered Galapagos petrel *Pterodroma phaeopygia* on San Cristóbal island: Conservation and management implications. Bird Conserv Int. 2010; 20: 306–319. doi: 10.1017/S095927091000002X

[69] Scott D, Moller H, Fletcher D, Newman J, Aryal J, Bragg C, et al. Predictive habitat modelling to estimate petrel breeding colony sizes: Sooty shearwaters (*Puffinus griseus*) and mottled petrels (*Pterodroma inexpectata*) on Whenua Hou Island. New Zeal J Zool. 2009; 36: 291–306. doi: 10.1080/03014220909510156

[70] Charleton K, Bragg C, Knight B, Fletcher D, Moller H, Newman J, et al. Spatial variation in burrow entrance density of the sooty shearwater (*Puffinus griseus*). Notornis. 2009; 56: 1–10.

[71] Schulz M, Robinson S, Gales R. Breeding of the Grey Petrel (*Procellaria cinerea*) on Macquarie Island: population size and nesting habitat. Emu—Austral Ornithol. 2005; 105: 323–329. doi: 10.1071/MU04058

[72] Mejías MA, Wingate DB, Madeiros JL, Wiersma YF, Robertson GJ. Nest-cavity selection and nesting success of Bermudian white-tailed Tropicbirds (*Phaethon lepturus catesbyi*). Wilson J Ornithol. 2017; 129: 586–599. doi: 10.1676/16-115.1

[73] Bourgeois K, Vidal É. Yelkouan shearwater nest-cavity selection and breeding success. Comptes Rendus—Biol. 2007; 330: 205–214. doi: 10.1016/j.crvi.2006.12.007 17434114

[74] Kulaszewicz I, Jakubas D. Influence of nest burrow microclimate on chick growth in a colonial High-Arctic seabird, the little auk. Polar Res. 2018; 37: 1547044. doi: 10.1080/17518369.2018.1547044

[75] Garcia-Borboroglu P, Yorio P. Effects of microhabitat preferences on kelp gull *Larus dominicanus* breeding performance. J Avian Biol. 2004; 35: 162–169. doi: 10.1111/j.0908-8857.2004.03149.x

[76] Fagundes AI, Ramos JA, Ramos U, Medeiros R, Paiva VH. Breeding biology of a winter-breeding procellariiform in the North Atlantic, the Macaronesian shearwater *Puffinus lherminieri baroli*. Zoology. 2016; 119: 421–429. doi: 10.1016/j.zool.2016.05.014 27353191

[77] Powell CDL, Wooller RD, Bradley JS. Breeding biology of the Flesh-footed Shearwater (*Puffinus carneipes*) on Woody Island, Western Australia. Emu—Austral Ornithol. 2007; 107: 275–283. doi: 10.1071/MU07005

[78] Cuthbert R, Davis LS. Adult survival and productivity of Hutton’s Shearwaters. Ibis (Lond 1859). 2002; 144: 423–432. doi: 10.1046/j.1474-919X.2002.00071.x

[79] Silva I, Menezes D, Oliveira P, Catry P, Granadeiro JP, Jardim CS, et al. Invasive Argentine ants prey on Bulwer’s petrels nestlings on the Desertas Islands (Madeira) but do not depress seabird breeding success. J Nat Conserv. 2018; 43: 35–38. doi: 10.1016/j.jnc.2018.02.013

[80] Rodríguez B, Siverio F, Acosta Y, Rodríguez A. Breeding success of Cory’s shearwater in relation to nest characteristics and predation by alien mammals. Ardeola. 2021; 69: 115–128. doi: 10.13157/arla.69.1.2022.sc1

[81] Price CA, Emery TJ, Hartmann K, Woehler EJ, Monash R, Hindell MA. Inter-annual and inter-colony variability in breeding performance of four colonies of short-tailed shearwaters. J Exp Mar Bio Ecol. 2021; 537: 151498. doi: 10.1016/j.jembe.2020.151498

[82] Bretagnolle V, Renaudet L, Villard P, Shirihai H, Carlile N, Priddel D. Status of Gould’s petrel *Pterodroma leucoptera caledonica* in New Caledonia: distribution, breeding biology, threats and conservation. Emu. 2021; 1–11. doi: 10.1080/01584197.2021.1938611

[83] Cuthbert R, Louw H, Lurling J, Parker GC, Rexer-Huver K, Sommer E, et al. Low burrow occupancy and breeding success of burrowing petrels at Gough Island: A consequence of mouse predation. Bird Conserv Int. 2013; 23: 113–124. doi: 10.1017/S0959270912000494

[84] Schreiber EA. Climate and weather effects on seabirds. Biology of marine birds. 2002. p. 179–216.

[85] Aubry LM, Koons DN, Monnat JY, Cam E. Consequences of recruitment decisions and heterogeneity on age-specific breeding success in a long-lived seabird. Ecology. 2009; 90: 2491–2502. doi: 10.1890/08-1475.1 19769127

[86] Daunt F, Wanless S, Harris MP, Money L, Monaghan P. Older and wiser: Improvements in breeding success are linked to better foraging performance in European shags. Funct Ecol. 2007; 21: 561–567. doi: 10.1111/j.1365-2435.2007.01260.x

[87] González-Solís J. Sexual size dimorphism in northern giant petrels: Ecological correlates and scaling. Oikos. 2004; 105: 247–254. doi: 10.1111/j.0030-1299.2004.12997.x

[88] Bester AJ, Priddel D, Klomp NI, Carlile N, O’Neill LE. Reproductive success of the Providence petrel *Pterodroma solandri* on Lord Howe Island, Australia. Mar Ornithol. 2007; 35: 21–28.

[89] Newman J, Fletcher D, Moller H, Bragg C, Scott D, McKechnie S. Estimates of productivity and detection probabilities of breeding attempts in the sooty shearwater (*Puffinus griseus*), a burrow-nesting petrel. Wildl Res. 2009; 36: 159–168. doi: 10.1071/WR06074

[90] Jones MJ. The relationship of chick-size and nest site occupancy with nest type and nesting density in Cory’s shearwater *Calonectrics diomedea* on Selvagem Grande. Bol Mus Mun Funchal. 1986; 38: 110–119.

[91] Pandolfi-Benoit M, Bretagnolle V. Seabirds of the southern lagoon of New Caledonia: Distribution, abundance and threats. Waterbird Soc. 2002; 25: 202–213. doi: 10.1675/1524-4695(2002)025[0202:SOTSLO]2.0.CO;2

[92] Olivier F, Wotherspoon SJ. Modelling habitat selection using presence-only data: Case study of a colonial hollow nesting bird, the snow petrel. Ecol Modell. 2006; 195: 187–204. doi: 10.1016/j.ecolmodel.2005.10.036

[93] Jones HP, Towns DR, Bodey TW, Miskelly C, Ellis JC, Rauzon MJ, et al. Recovery and restoration on seabird islands. Seabird islands: ecology, invasion, and restoration. 2011. p. 317–357. doi: 10.1093/acprof:osobl/9780199735693.003.0011

[94] Bolton M, Medeiros R, Hothersall B, Campos A. The use of artificial breeding chambers as a conservation measure for cavity-nesting procellariiform seabirds: A case study of the Madeiran storm petrel (*Oceanodroma castro*). Biol Conserv. 2004; 116: 73–80. doi: 10.1016/S0006-3207(03)00178-2

