# Supplementary material for: Nest-site selection and its influence on breeding success in a poorly-known and declining seabird: The Tahiti petrel Pseudobulweria rostrata
Source: PLoS One. 2022 Apr 27;17(4):e0267408. doi: 10.1371/journal.pone.0267408 (PMC9045628; doi:10.1371/journal.pone.0267408)

S1 Fig: Map of the quadrats distribution on Nemou Island.

The map shows (i) the position of quadrats whitin and outisde Tahiti petrel (TP) nesting habitat, (ii) the location of the edge of the TP sub colonies, and (iii) the vegetation classification. Quadrats are shown to scale.
The black section (Anthropized area) is the area where vegetation was cut out to set up a camping area

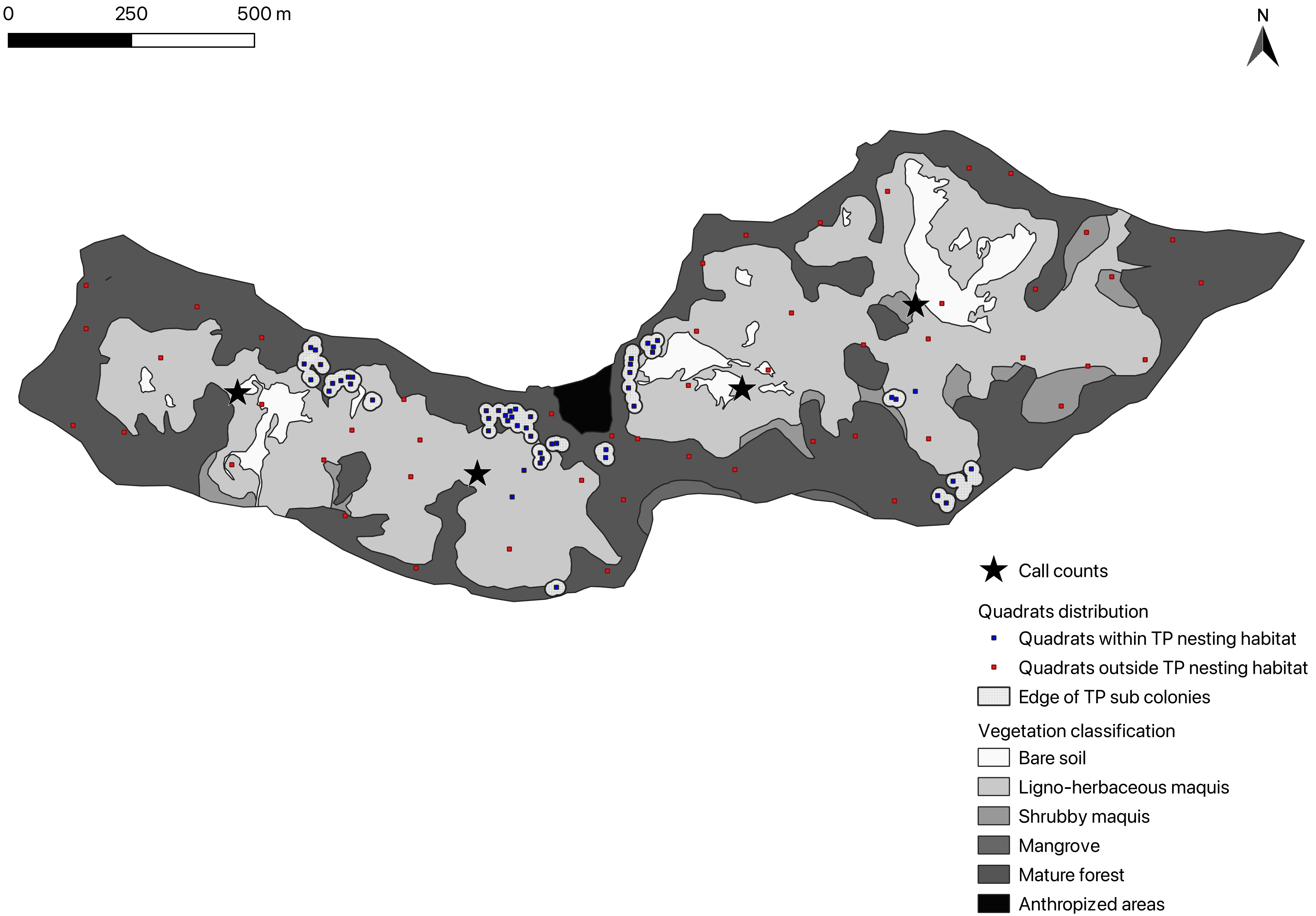

Supplement: S1 Fig — The map shows (i) the position of quadrats whitin and outisde Tahiti petrel (TP) nesting habitat, (ii) the location of the edge of the TP sub colonies, and (iii) the vegetation classification. Quadrats are shown to scale. The black section (Anthropized area) is the area where vegetation was cut out to set up a camping area. (PDF) [file pone.0267408.s001.pdf]
