# Supplementary material for: Nest-site selection and its influence on breeding success in a poorly-known and declining seabird: The Tahiti petrel Pseudobulweria rostrata
Source: PLoS One. 2022 Apr 27;17(4):e0267408. doi: 10.1371/journal.pone.0267408 (PMC9045628; doi:10.1371/journal.pone.0267408)
Supplement: S1 Table — (PDF) [file pone.0267408.s002.pdf]

S1 Table: Predictive habitat models selection for Tahiti petrel based upon corrected Akaike's Information Criterion. Only models with ΔAICc < 2 were shown.

Models		AIC[1]	ΔAICc[2]	wAICc[3]
H1	Cos + Sin + S + HG + SD + DC	99.7	0.00	0.63
H2	Cos + Sin + S + HG + SD	100.7	1.05	0.37

Cos = cosinus of the orientation variable, Sin = Sinus of the orientation variable, S = Slope , HG = Habitat groups from the clustering (i.e., G1 = Open habitat; G2 = Closed habitats; G3 = Rocky habitats), SD = Soil depth, DC = Distance to coastline

[1] Value of the Akaike Information Criterion for limited sample sizes.
[2] Difference between the AICc of each model and the AICc of model 1 (H1).
[3] Normalized Akaike weight which can be interpreted as the proportion of support in the data for a given model.
