# Supplementary material for: Nest-site selection and its influence on breeding success in a poorly-known and declining seabird: The Tahiti petrel Pseudobulweria rostrata
Source: PLoS One. 2022 Apr 27;17(4):e0267408. doi: 10.1371/journal.pone.0267408 (PMC9045628; doi:10.1371/journal.pone.0267408)
Supplement: S2 Table — (PDF) [file pone.0267408.s003.pdf]

S2 Table: Models statistic used to predict the influence of burrow characteristics and environmental variables on the Tahiti petrel breeding success. Only models with ΔAICc < 2 were shown.

Models		AICc[1]	ΔAICc[2]	wAICc[3]
B1	L + D + T	116.24	0.00	0.50
B2	L + T	117.39	1.14	0.28

L = entrance width of the burrow, D = burrow depth, T = Type of burrow (i.e., root (BB), soil (BS), rocky (C)).

[1] Value of the Akaike Information Criterion for limited sample sizes.
[2] Difference between the AICc of each model and the AICc of model 1 (B1).
[3] Normalized Akaike weight which can be interpreted as the proportion of support in the data for a given model.
